# Heredity

**Published:** 1889-10-26

**Authors:** 


### HEREDITY.

In an article headed "Heredity,"‡ Sir William Turner, of Edinburgh, remarks, "the subject is in the air at the present time." It is, however, by no means a new subject, as it has been discussed ever since the days of Aristotle. That certain diseases are hereditary was recognised by Hippocrates. The first aspect of the question is whether any physical basis can be found for heredity. There is the important fact that the young animal arises by the fusion of an extremely minute particle derived from either parent. There is, therefore, a physical continuity, and this carries with it certain properties which cause the offspring to reproduce not only bodily configuration, but also other properties. Had one been discussing heredity disease twenty years ago, tuberculosis would have been adduced as a prominent example, but the progress of knowledge of late years tends to throw some doubt on this. Yet, if it be allowed that tubercle arises from a bacillus, it would appear that some particular constitution which renders liable to the attack of bacillus is transmitted. Similarly with cancer, which has recently been attributed to a bacillus. Again, heredity is undoubtedly one of the most powerful factors in the production of gout and rheumatism, as shown by Sir Dyce Duckworth. Again, some families exhibit a remarkable tendency to hæmophilia, also to cataract. If it cannot be said that the structural lesion is transmitted, there can be no doubt that the tendency or predisposition is transmitted. When, however, we speak of tendencies, or proclivities, or susceptibilities, we employ terms which are somewhat vague, as we are unable to recognise in the plasma any structural change which would enable us to say that a particular tendency will be manifested. Still, it is none the less certain that these terms express an important truth. This, however, is but a part of the subject of heredity. There is no doubt that characters which have been acquired by the parent may be transmitted. For example, a mutilation which has affected a parent has been transmitted to offspring. Impressions made on the mother being reproduced in the offspring has been patent since Jacob set peeled rods before the flocks in order to influence the colour and marking of the young. We have in recollection the case of a child born with one hand only, the other being absent and the forearm resembling a stump; and we also know that the mother of this child was alarmed, some time previously, by a beggar thrusting the stump of his arm before her face.

‡ *Medical Press and Circular*, Oct. 2, 1889.

